# Influence of social distancing on physical activity among the middle-aged to older population: Evidence from the nationally representative survey in China

**DOI:** 10.3389/fpubh.2022.958189

**Published:** 2022-11-04

**Authors:** Wuping Zhou, Lanyue Zhang, Ting Wang, Qiaosheng Li, Weiyan Jian

**Affiliations:** Department of Health Policy and Management, School of Public Health, Peking University, Beijing, China

**Keywords:** physical activity, social distancing, middle-aged to older population, health promotion, health education, China

## Abstract

**Background:**

Group-based physical activity is an important positive factor assisting the middle-aged to older population to be regularly physically active, especially inside a society with a large population and highly sociable environment. However, when group-based physical activity is restricted during a public health crisis such as the infectious disease pandemic, the influence of social distancing on physical activity among this vulnerable group needs to be recognized.

**Objectives:**

This study aimed to investigate the influence of social distancing on physical activity among the middle-aged to older Chinese population at the national level.

**Methods:**

Data from a nationally representative social follow-up survey (China Family Panel Studies, CFPS) for 2018 and 2020 were used. Physical activity level in year 2018 was set as the baseline to be compared with that for each individual in 2020, when China implemented social distancing during the COVID-19. Chinese population with an age greater than 45 years were included, and three levels of physical activity were established. Logistic models were developed to identify sociodemographic characteristic that may be associated with a higher probability of worse PA behaviors during the social distancing.

**Results:**

Over 46% respondents could be described as being Physically Inactivity during 2018 and this proportion increased to 67.2% in 2020. Respondents who live in the Northeast or rural regions, having a spouse, being employed, having a low level of education, and being of low-income level showed a higher decrease in physical activity compared to other groups. However, individuals living with chronic diseases emerge as being more likely to maintain positive habits with respect to physical activity in this context.

**Conclusion:**

Social distancing during the COVID-19 pandemic has significantly influenced the extent of physical activity among middle-aged to older Chinese residents. This is especially true in respect to middle-aged and elderly people who are at increased risk of chronic diseases. Given this, there is a clear need to consider effective modalities for physical activity in the context of social distancing based on home quarantine and city lockdown. Furthermore, specific health-related strategies need to be considered in relation to different regions and populations.

## Introduction

The promotion of physical activity is becoming increasingly important among the middle-aged to older population, especially for those who live with chronic diseases. Based on the social character of participation, group-based sports are associated with better health outcomes compared to individual activities (1). Moreover, peer-support interventions were also shown to be effective at increasing older people's physical activity levels (2–4). Evidence from previous studies has shown that social support could improve physical activity in the elderly population, and compared with family members, the most useful form of social support appeared to come from others, such as receiving suggestions from health professionals, and getting demonstrations from exercise experts (5, 6).

However, the COVID-19 pandemic has given rise to an unprecedented public health crisis, with many of its implications being life-altering. Across the world, to limit the spread of COVID-19, stringent restrictions have been imposed on social contact, often achieved through the implementation of social distancing and home quarantines. Although better access to exercise parks and facilities had been found to be related to a greater amount of physical activity among older adults (7, 8), both indoor and outdoor activity facilities were closed due to the city-wide lockdown in many countries during the pandemic.

Therefore, these restrictive measures resulted in lifestyle changes, such as increased sedentary behavior, reduction of physical activities, and other unhealthy behaviors. Physical inactivity is one of the leading risk factors for chronic disease mortality, often causing the premature deterioration of health in humans. Regular daily exercise can improve individuals' health by strengthening their immune systems and counteracting certain co-morbidities such as obesity, diabetes, and serious heart conditions that make individuals more susceptible to severe COVID-19 illness (9). In addition, performing physical activities during the COVID-19 pandemic is also associated with lower levels of depressive experience and anxiety (10). However, evidence in existing studies have identified that COVID-19 home quarantine with social distancing had a negative effect on physical activities (11–16).

In 2020, to tackle the COVID-19 pandemic, China implemented aggressive strategies with respect to social prevention and containment (17). However, the most common physical activities among the middle-aged to older population in China are traditional Chinese sports, such as Tai Ji Quan and Yangko exercises (also called social dancing or square-dancing) (18–20). These two kinds of physical exercise are usually practiced in groups in community centers or parks and develop social interaction among participants at the same time (21–23). Therefore, there is a clear need to consider changes in physical activity among Chinese middle-aged to older populations during the quarantine time. However, few existing studies have focused on the matter of changes in physical activity across the period before and during the pandemic among middle-aged to older Chinese populations at the national level. Moreover, most of the available studies on the impact of social distancing on physical activity have considered only small samples using online surveys. It is on this basis that the present study considers Chinese nationally representative data to study the influence of social distancing on physical activity. Given that the middle-aged to older population constitutes a high-risk group with respect to chronic diseases, it is this population that is considered in relation to this matter.

Based on the existing research, this study assumes that the lock down may affect the physical activity of the middle-aged to older population in China to a large extent. For different subgroups of the middle-aged to older residents, the degree of influence is not completely consistent. Those with higher social status and higher education level have a higher awareness of self-care and thus are more likely to maintain good exercise habits during the pandemic. Identifying vulnerable groups may help promote physical activity during the pandemic.

## Materials and methods

### Material

This study used data from the China Family Panel Studies (CFPS), which is a nationally representative, comprehensive, longitudinal social survey of the Chinese population. The database covered a wide range of topics and included integrated modules for education, regions, health, and other information (24). To ensure quality among the data, CFPS made use of multistage and stratified random sampling methods. First, 25 provinces in China were divided into five sampling frames, samples of each sub-sample frame are extracted in three stages: administrative district/county, administrative village/committee, and household. In the first two stages of sampling, the official administrative division data is used. In the third stage, the map address method is used to build a sampling box, and the cyclic equidistant sampling method with random starting points is used to sample households.

Strict quality control protocols are in force at the stage at which the project is implemented, and database construction is performed professionally. CFPS surveys are conducted every 2 years, with the most recent once being conducted in 2020. The time of data collection were Jun 2018 to Mar 2019 and Jun 2020 to Mar 2021. The Biomedical Ethics Review Committee of Peking University approved CFPS, and all participants were required to provide written informed consent. The ethical approval number was IRB00001052-14010.

### Sample

Each respondent was assigned a unique ID code that is consistent from year to year, making it possible for the present study to be conducted with panel data spanning multiple years. According to the age classification from the “National Standard for Physical Exercises Guideline 2020” by the General Administration of Sport of China, the sample group for this study comprised participants aged 45 years and older, all of whom were surveyed by CFPS in 2018 and 2020, with a sample size of 9,763 cases each year (25).

### Dependent variables of physical activities

In the CFPS, the respondents were asked how often and how long they participated in physical activities on average per week. The responses to these questions were grouped into the following categories: never participated in physical activities; participated but less than once per month on average; once or twice a week on average; three to four times a week on average; five times a week or more. All the respondents not falling into the category of the first group “never participated in physical activities” were asked “how many minutes of physical activity at a time”, and the specific duration (in minutes) would then be noted by the respondents.

The “Healthy China Action” blueprint advises individuals to engaged in three times of physical activity per week, with each lasting at least 30 min (26, 27). According to the recommended physical activity standards in this blueprint, we divided physical exercise habits into the following three categories: physical inactivity (less than once a week); regular physical activity (more than three times a week, each time lasting longer than 30 min); irregular physical activity (other cases). This classification has also been used in the previous study (28).

The dependent variable in this study was the change in physical activity habits during the period of social distancing in 2020, compared with that in 2018. In detail: For the physically inactive subset at baseline (2018), the criterion is whether the given individuals engaged in greater levels of physical activity (1 if the frequency of physical activities in 2020 is not 0; 0 otherwise). For the subset of irregular physical activity at baseline, the criterion is whether they engaged in a lower level of physical activity (1 if the frequency of physical activities in 2020 is 0; 0 otherwise). Finally, for the subset of regular physical activity at baseline, the criterion is whether they engaged in less frequent physical activity (1 if the given individual did not reach the regular physical activity in 2020; 0 otherwise).

### Independent variables

Social economic status variables were used as control variables, including the respondent's personal characteristics, social status, health status, location, etc., and in the manner evident in the classification of these variables in the existing literature (19, 29). The specific rules governing this classification are provided in Table 1.

**Table 1:** Recode rules regarding independent variables.

**Variables**	**Definition**	**Assignment**
Region	Multiple classes of dummy variables: Jiangsu Province, Zhejiang Province, etc.	Four classes of dummy variables^*^: 0 = West: Xinjiang Uygur Autonomous Region, Tibet Autonomous Region, Ningxia Hui Autonomous Region, Inner Mongolia Autonomous Region, Gansu Province, Qinghai Province, Shaanxi Province, Yunnan Province, Sichuan Province, Guizhou Province, Chongqing Municipality, Guangxi Zhuang Autonomous Region, 1 = Middle: Shanxi Province, Henan Province, Anhui Province, Hubei Province, Hunan Province, Jiangxi Province 2 = East: Beijing, Tianjin, Shanghai, Hebei Province, Shandong Province, Zhejiang Province, Jiangsu Province, Guangdong Province, Hainan Province 3 = Northeast: Heilongjiang Province, Jilin Province, Liaoning Province
Urban/rural	Two classes of dummy variables: Urban/Rural	Two classes of dummy variables: Urban/Rural; 0 = Urban, 1= Rural
Gender	Two classes of dummy variables: Male/Female	Two classes of dummy variables: Male/Female; 0 = Male, 1= Female
Age	Continuous variables range over 45 years old	Three classes of dummy variables: 0 = Middle-aged individuals range from 45 to 59 1 = Early elderly individuals range from 60 to 74 2 = Late elderly individuals range over 75
Marital status	Five classes of dummy variables: Unmarried, Married, Cohabiting, Divorced, Widowed	Two classes of dummy variables: 0 = With spouse at present: Married, Cohabiting 1 = No spouse at present: Unmarried, Divorced, Widowed
Work status	Two classes of dummy variables: In working condition, Not in working state (retired)	Two classes of dummy variables: 0 = In working condition, 1 = Not in working state (retired)
Education level	Eight classes of dummy variables: Illiterate, Kindergarten, Elementary school, Junior high school, High school, College, Bachelor's degree, Master's degree, Doctorate	Three class of dummy variables: 0 = Primary Education: Illiterate, Kindergarten, Elementary school, 1 = Secondary Education: Junior high school, High school, 2 = Higher Education: College, Bachelor's degree, Master's degree, Doctorate
Self-assessed income level	Five classes of dummy variables: High-income, Upper-Middle-income, Middle-income, Lower-Middle-income, Low-income	Five classes dummy variables: 0 = High-income, 1 = Upper-Middle-income, 2 = Middle-income, 3 = Lower-Middle-income, 4 = Low-income
Chronic diseases	Two classes of dummy variables: with chronic diseases/without chronic diseases	Two classes dummy variables: 0 = With chronic diseases, 1 = Without chronic diseases
Self-assessed health status	Five classes of dummy variables: Excellent; Very good; Good; Fair; Poor	Five classes dummy variables: 0 = Excellent; 1 = Very good; 2 = Good; 3 = Fair; 4 = Poor

* Provinces are located in regions in accordance with the divisions published by the National Bureau of Statistics of the People's Republic of China.

### Statistics analysis

We established two timepoints: (1) 2018 baseline; (2) during COVID-19 social distancing in 2020. The baseline represents the state of physical activity before COVID-19 social distancing, and the year of 2020 represents the state during the COVID-19 social distancing. A logistic regression was constructed below to ascertain the factors associated with changes in physical activity during social distancing, especially in the case of individuals with different physical activity at the baseline. Formula 1 shows the model settings:


$$\begin{array}{l} ln\frac{P}{1-P}=\beta_{0}+\beta_{1}X_{1}+\beta_{2}X_{2}+\ldots +\beta_{i}X_{i}+\varepsilon \; \end{array}$$

Where the P is the probability of physical activity behavior change as described in the Materials and Methods Section (previously physically inactive population engaged in higher levels of physical activity; previously irregular physical activity population developed worse habits regarding physical activity; previously regular physical activity population developed worse habits regarding physical activity). X_i_ represents the social-economic status such as age, gender. Heteroskedastic robust standard errors were calculated. The OR and the *P*-value are presented in the Results Section.

The significant level α in this study was set at 0.05 and the statistical analysis was carried out using Stata Version 16.0 (Stata/SE, StataCorp LLC, TX, USA).

## Results

### Sample characteristics

The characteristics of the samples in this study are summarized in Table 2. Middle-aged adults (45–59) accounted for more than half of the sample, followed by the early elderly group (60–74), accounting for 39.3%, and then the late elderly group (over 75 years), accounting for only 6.9%. 88.5% respondents of the sample reported having spouses, and 69.9% of the respondents reported having jobs. About 55.5% of respondents of the sample reported having received primary education, with the proportion with secondary education accounting for 43.2%. In terms of income level, 44.4% of the respondents reported that they were in the middle-income group, and 14.0% respondents reported income levels placing them in the high-income group. 76.1% of respondents had one or more chronic diseases.

**Table 2:** Demographic-related and other characteristics of middle-aged to older participants in CFPS (2020).

**Characteristics**	***N***	**%**
**Region**		
Northeast	1,549	15.9
East	3,260	33.4
West	2,563	26.3
Central	2,391	24.5
**Rural/urban**		
Rural	5,058	51.8
Urban	4,705	48.2
**Gender**		
Female	4,815	49.3
Male	4,948	50.7
**Age**		
Middle aged (45–59)	5,247	53.7
Early elderly adult (60–74)	3,839	39.3
Late elderly adult (75-)	677	6.9
**Marital status**		
Have spouse	8,645	88.5
No spouse	1,118	11.5
**Work status**		
None	2,942	30.1
Yes	6,821	69.9
**Education level**		
Primary education	5,417	55.5
Secondary education	4,216	43.2
Higher education	130	1.3
**Income level**		
Low	996	10.2
Low-middle	1,611	16.5
Middle	4,334	44.4
Upper-middle	1,456	14.9
High	1,366	14.0
**Chronic disease**		
None	7,428	76.1
Yes	2,335	23.9
**Self-assessed health status**		
Excellent	1,191	12.2
Very good	1,135	11.6
Good	3,844	39.4
Fair	1,442	14.8
Poor	2,151	22.0

### Physical activity before and during the COVID-19 social distancing

Table 3 displays data relating to physical activity among the middle-aged to older Chinese individuals during 2018 and 2020. 46.4% were physical inactivity at baseline, with the proportion increased to 67.2% during the social distancing. The proportion of irregular physical activity decreased from 18.2% in 2018 to 17.1% in 2020. Meanwhile, 35.5% of the respondents reported exercising regularly at baseline, but only 15.7% reported maintaining their habit of engaging in regular exercise during the social distancing. Compared to 2018, the surveyed year preceding the social distancing of COVID-19, the decrease of physical activity emerged as statistically significant for 2020 (*P* < 0.001).

**Table 3:** Physical activity among the middle-aged to older Chinese population (2018 and 2020).

**Level of physical activity**	**2018**		**2020**		**P**
	***N***	**%**	***N***	**%**	
Physical inactivity	4,525	46.4	6,565	67.2	
Irregular physical activity	1,775	18.2	1,669	17.1	<0.001
Regular physical activity	3,463	35.5	1,529	15.7	
Total	9,763	100.0	9,763	100.0	

### Results of multiple logistic regression on changes in physical activity

From a regional perspective, it emerges that it is more likely that the physical inactivity individuals who started performing physical activities during social distancing were from Central (OR 1.37, *P* < 0.01) and Western China (OR 1.44, *P* < 0.01). However, in Northeast China, people who used to be irregular physically active were 1.35 times more likely to reduce their physical activities than those in the Eastern region.

Living in a rural area is clearly another risk factor. The rural residents who used to engage in regular physical activity emerged as being 2.16 times more likely to have reduced their physical exercises during the social distancing period than those in the urban area. The probability of people engaged in irregular physical exercise reducing their physical activity level was 1.65 times higher than those in urban areas. At the same time, the urban residents who did not exercise before social distancing were 1.89 times more likely to increase their participation in physical activity than the rural residents.

Those without a spouse or unemployed who did not exercise before the social distancing were more likely to participate in physical activities during the social distancing period (OR, 1.3 and 1.75, respectively). And those noted as working and as having an exercising habit were more likely to reduce their participation in physical activities (OR, regular exercise 1.6, irregular exercise 1.35).

Education is an important protective factor. Compared with those noted as having received only primary education, the group whose members received secondary or higher education were associated with a higher probability of engaging in increased physical activities and a lower probability of engaging in reduced physical activities. Additionally, middle-income individuals who did not participate in physical activities before the COVID-19 social distancing were more likely to perform physical activity than those in the low-income group, as well as being less likely to reduce their levels of physical activity.

People with chronic diseases were more likely to maintain good physical exercise habits than those without. After controlling for other variables, the effects of gender and age on physical activity change during social distancing among the middle-aged to older Chinese people were not significant.

Multiple logistic regression results on changes in physical activity are presented in Table 4.

**Table 4:** Multiple logistic regression results changes in physical activity during COVID-19 social distancing among people with different physical activity at baseline (OR).

**Characteristics**	**Model 1 Physical inactivity in 2018**		**Model 2 Irregular physical activity in 2018**		**Model 3 Regular physical activity in 2018**	
	**OR (95% CI)**	***P***	**OR (95% CI)**	***P***	**OR (95% CI)**	***P***
**Regions**						
East (ref.)	—		—		—	
Northeast	0.88 (0.67, 1.16)	0.374	1.35 (1.01, 1.81)	0.040	1.24 (0.98, 1.56)	0.074
Central	1.37 (1.11, 1.69)	0.003	0.94 (0.74, 1.20)	0.643	0.83 (0.67, 1.02)	0.074
West	1.44 (1.16, 1.78)	0.001	1.03 (0.80, 1.33)	0.810	1.13 (0.90, 1.40)	0.287
**Urban/rural**						
Urban (ref.)	—		—		—	
Rural	0.53 (0.45, 0.62)	<0.001	1.65 (1.34, 2.03)	<0.001	2.16 (1.79, 2.60)	<0.001
**Gender**						
Male (ref.)	—		—		—	
Female	1.16 (0.98, 1.37)	0.085	1.05 (0.86, 1.28)	0.626	0.87 (0.74, 1.03)	0.114
**Age**						
Middle age (45–59) (ref.)	—		—		—	
Early elderly adult (60–74)	1.09 (0.92, 1.31)	0.322	1.04 (0.84, 1.29)	0.716	0.99 (0.83, 1.19)	0.917
Late elderly adult (75-)	0.82 (0.56, 1.22)	0.329	1.12 (0.69, 1.82)	0.659	1.38 (1.00, 1.90)	0.052
**Marital status**						
With spouse (ref.)	—		—		—	
No spouse	1.30 (1.02, 1.67)	0.035	1.10 (0.79, 1.52)	0.564	0.87 (0.68, 1.12)	0.292
**Work status**						
Not working (ref.)	—		—		—	
Working	0.57 (0.46, 0.69)	<0.001	1.35 (1.07, 1.71)	0.013	1.60 (1.33, 1.94)	<0.001
**Education level**						
Primary (ref.)	—		—		—	
Secondary	1.68 (1.42, 2.00)	<0.001	0.54 (0.43, 0.66)	<0.001	0.58 (0.48, 0.69)	<0.001
Higher education	3.55 (1.57, 7.99)	0.002	0.24 (0.14, 0.39)	<0.001	0.46 (0.27, 0.78)	0.004
**Self-assessed income level**						
Low Income (ref.)	—		—		—	
Low-middle income	1.47 (1.06, 2.03)	0.020	1.31 (0.89, 1.93)	0.175	0.74 (0.53, 1.03)	0.072
Middle income	1.37 (1.02, 1.84)	0.036	1.21 (0.84, 1.74)	0.308	0.67 (0.50, 0.90)	0.008
Upper-middle income	1.48 (1.05, 2.08)	0.026	1.29 (0.86, 1.95)	0.220	0.61 (0.44, 0.86)	0.004
High income	1.39 (0.98, 1.97)	0.068	1.74 (1.12, 2.71)	0.014	0.72 (0.50, 1.03)	0.069
**Chronic disease**						
With chronic disease (ref.)	—		—		—	
Without chronic disease	1.17 (0.96, 1.44)	0.122	0.69 (0.54, 0.87)	0.002	0.87 (0.71, 1.05)	0.145
**Self-assessed health status**						
Excellent (ref.)	—		—		—	
Very good	0.77 (0.55, 1.08)	0.072	0.85 (0.58, 1.24)	0.397	1.02 (0.73, 1.44)	0.890
Good	0.95 (0.73, 1.23)	0.184	0.77 (0.57, 1.05)	0.100	0.82 (0.62, 1.09)	0.170
Fair	0.81 (0.59, 1.11)	0.685	0.98 (0.67, 1.43)	0.902	1.04 (0.74, 1.45)	0.830
Poor	0.76 (0.56, 1.03)	0.132	1.25 (0.85, 1.84)	0.249	1.28 (0.92, 1.78)	0.146

Model 1 demonstrated whether individuals who were physical inactivity in 2018, and increased their physical activity during the social distancing (χ^2^ = 189, *P* < 0.001, *Pseudo R*^2^ = 0.0511, *Degree of freedom* = 4, 446); Model 2 demonstrated whether individuals engaged in irregular physical activity in 2018 but subsequently became physical inactivity (χ^2^ = 151, *P* < 0.001, *Pseudo R*^2^ = 0.0483, *Degree of freedom* = 1, 761); Model 3 demonstrated whether individuals engaged in regular physical activity in 2018 but decreased physical activity in 2020 (χ^2^ = 264, *P* < 0.001, *Pseudo R*^2^ = 0.0745, *Degree of freedom* = 3, 425).

## Discussion

In this study, we analyzed the influence of social distancing on physical activity among the middle-aged to older Chinese population during the COVID-19 pandemic by using nationally representative samples. We followed-up and monitored the physical activity status of participants before and during the social distancing of the COVID-19 pandemic. In general, we found that physical activity has significantly decreased during the COVID-19 pandemic, with social distancing reducing to a significant extent residents' engagement in physical activity. Such reduction in physical activity levels in China is consistent with the findings of previous studies in other countries. However, we also found significant differences in terms of the effect of social distancing on physical activity across regions and sociodemographic characteristics.

From the regional perspective, levels of physical activity were more severely affected in Northeast China, a result consistent with observations made in a previous study (30). In the coldest climate regions in China, the closure of winter sports facilities based on social distancing rules in the Northeast offers a straightforward explanation for the increased levels of physical inactivity among the residents.

We found that the decrease in physical activity of rural residents was more significant during the social distancing period. This result was inconsistent with previous studies conducted in other countries (31–34). In addition, as the ability of individuals to access, process, and understand health information to make decisions, health literacy is extremely important for population health within the social distancing context (35). Considering that the lack of health-related knowledge and lower levels of health literacy are common among the elderly in rural areas as compared with their urban counterparts in China, this provides another potential reason for physical activity reduction among the rural elderly population during the COVID-19 social distancing period (36, 37).

Our study highlights that having a spouse, being employed, having a lower level of education, and having a lower level of income are all related with a higher probability of having negative habits during the pandemic. Having chronic diseases appears to be associated with a higher probability of maintaining more positive habits of physical activity.

It is also the finding of our study that the groups characterized by low levels of education or low levels of income are associated with a higher probability of decreasing physical activity. This result was consistent with previous literature (38, 39). Employed individuals emerge as being more likely to reduce their physical activities, this may be because those who are currently employed might have reduced their commuting-related physical activities due to their employment occurring within their own homes (40).

We unexpectedly found that people with high levels of income emerged as being associated with a failure to maintain positive physical activity habits during the period of social distancing. According to the previous studies, the middle-aged group with a high level of income was the main group of fitness centers consumption, particularly in the high-level income population; they prefer to pursue more professional and personalized one-to-one “private services” (23, 41). By way of an explanation for this stage of affairs, it might be the case that high earners were used to exercising in high-end fitness centers and the locking down for social distancing of such centers led to a disruption in such individuals' exercise habits.

After controlling for gender and age, we proposed that chronic disease played a protective role in physical activity. This finding was inconsistent with previous study findings that people who have lifestyle-related chronic conditions such as diabetes and high blood pressure have been less active during the pandemic than those without such chronic diseases (42). These results may relate to certain health-related promotional policies in China in recent years, including health education in accordance with the characteristics of different target groups within the population (26). In fact, doctors will provide targeted education to patients who are diagnosed with chronic diseases, and physical activity is a common health education prescription (26). In addition, the proportion of the Entire-population Family Doctors Service Contracts rose from 28.33% in 2015 increased to 75.46% in 2020 (43). Such an increase in the proportion of family doctors' coverage could have led to greater access to health education and exercise prescription among middle-aged to older people, especially those with chronic diseases.

There were several limitations in the current study that must be acknowledged. First, due to certain data-related constraints, only data relating to frequency and duration of physical activity were consulted, making it impossible to identify what kind of physical activities might be associated with a stronger impact on residents' exercise. Furthermore, regarding the observation that people who are physically inactivity in daily life with no spouse increased their physical exercise during the social distancing period, due to the limitation of the data, we were not able to conduct a deeper exploration of the characteristics of this group, nor to formulate an explanation for this pattern. This finding needs further research.

## Conclusion

To control the spread of the infectious virus, restrictions such as social distancing and lockdowns have been imposed worldwide, and so there is a need to examine the consequences changes in living environment and the limiting of lifestyles options has imposed. As with the health education programs that have been provided during the pandemic period, there is also a need for remote network services such as in-home physical activity tutorials and online fitness coaching courses. Our findings reported here suggest that in order to maintain healthy behaviors during social distancing, there is a specific need to develop effective strategies for the promotion of physical activity that targets members of vulnerable populations when quarantine or restriction approaches are implemented.

## Data availability statement

Publicly available datasets were analyzed in this study. This data can be found here: CFPS repository: http://www.isss.pku.edu.cn/cfps/.

## Ethics statement

The studies involving human participants were reviewed and approved by the Biomedical Ethics Review Committee of Peking University approved CFPS, and all participants were required to provide written informed consent. The ethical approval number was IRB00001052-14010. The patients/participants provided their written informed consent to participate in this study.

## Author contributions

WZ conceived the study, performed the data compilation and formal data analysis, interpreted the findings, and wrote the manuscript. LZ conceptualized, drafted, and wrote the manuscript and interpreted the findings. TW contributed to the literature search and interpretation. QL performed the data analysis. WJ conceived of the study and participated in its design and coordination and helped to draft the manuscript. All authors have read and approved the final version of the manuscript and agree with the order of presentation of the authors.

## Funding

This work received funding from the National Natural Science Foundation of China (Grant Number: 71774003). The funder had no role in the study design, data collection, data analysis, data interpretation, or writing of the report.

## Conflict of interest

The authors declare that the research was conducted in the absence of any commercial or financial relationships that could be construed as a potential conflict of interest.

## Publisher's note

All claims expressed in this article are solely those of the authors and do not necessarily represent those of their affiliated organizations, or those of the publisher, the editors and the reviewers. Any product that may be evaluated in this article, or claim that may be made by its manufacturer, is not guaranteed or endorsed by the publisher.
